# A rare case of a retroperitoneal enterogenous cyst with in-situ adenocarcinoma

**DOI:** 10.1186/1477-7819-5-113

**Published:** 2007-10-10

**Authors:** Jeffrey T Lordan, Robin L Jones, Nariman D Karanjia, Stefano de Sanctis, James G Woodland, Gary Middleton, Neville Menezes

**Affiliations:** 1 Department of Surgery, Royal Surrey County Hospital, Guildford, Surrey. GU2 7XX, UK; 2 Department of Oncology, Royal Surrey County Hospital, Guildford, Surrey. GU2 7XX, UK; 3 Department of Histopathology, Royal Surrey County Hospital, Guildford, Surrey. GU2 7XX, UK; 4 Hepatobiliary surgery, St. Peter's Hospital, Guildford Road, Chertsey, Surrey, KT16 0PZ, UK

## Abstract

**Background:**

Retroperitoneal enterogenous cysts are uncommon and adenocarcinoma within such cysts is a rare complication.

**Case presentation:**

We present the third described case of a retroperitoneal enterogenous cyst with adenocarcinomatous changes and only the second reported case whereby the cyst was not arising from any anatomical structure.

**Conclusion:**

This case demonstrates the difficulties in making a diagnosis as well as the importance of a multi-disciplinary approach, and raises further questions regarding post-operative treatment with chemotherapy.

## Background

We present a patient with a retroperitoneal enterogenous cyst that contained adenocarcinoma cells presenting in adulthood. To date, only 3 cases have been reported of retroperitoneal enterogenous cysts [1-3] and only a further 2 cases of such cysts developing adenocarcinomatous changes[4,5].

## Case presentation

**Figure 1:**
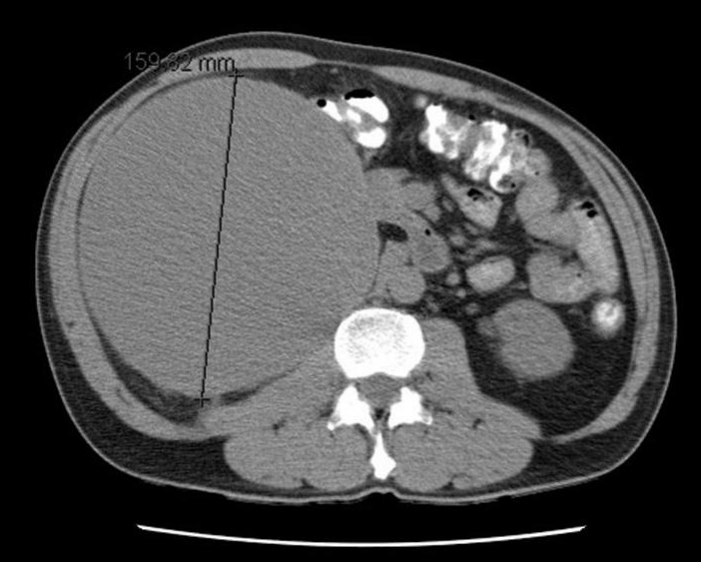
Computed tomography of the abdomen showing large mass lesion.

In 2002, a 54 year old man presented with mild heart-burn. Incidental "hepatomegaly" was found on examination accompanied by a slightly elevated bilirubin but otherwise normal liver function. A computerized tomography (CT) scan reported a well defined cyst arising from the right lower pole of the liver, measuring 14 × 12 × 10 cm (figure 1). Hydatid was excluded and the cyst was thought to be simple and benign, due to the absence of calcification. It was decided to treat this conservatively and the patient was discharged.

Four years later, he re-presented with a 5-month history of intermittent dull right sided abdominal pain and a right sided palpable abdominal mass was palpated. Liver function tests revealed a bilirubin of 29 but no other abnormalities. His α-feto-protein was 3 and CA 19.9 was 22129.

The repeat CT scan concurred with the previous scan, reporting a large benign cyst, with a maximum diameter of 16 cm. Figure 1 demonstrates the CT with the cyst, and the marker is the measure of the maximum diameter. Endoscopic ultrasound demonstrated a frond-like mucosal prominence within the cyst wall lining.

At laparotomy, the cyst was found to be retroperitoneal lateral to the right psoas muscle, displacing the pancreas and duodenum medially, the liver superiorly and the right kidney and ureter inferiorly. It did not appear to be arising from any particular organ. An attempt at complete excision was successful but there was some spillage of viscous cyst fluid. The abdominal cavity was thoroughly lavaged with water, followed by saline.

**Figure 2:**
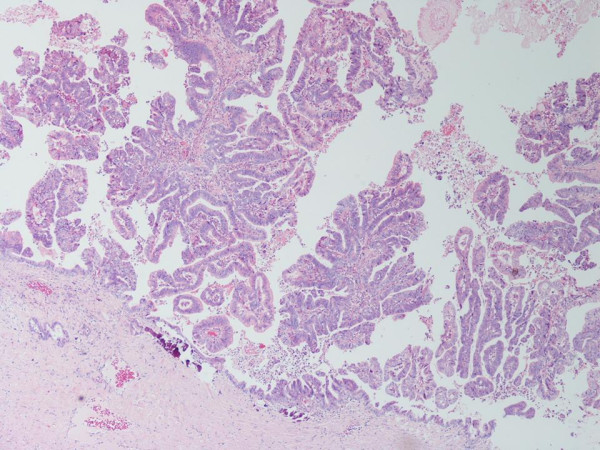
Photomicrograph of the lesion showing invasive mucinous cystadenocarcinoma (H and E ×400).

Histology reported an invasive mucinous cystadenocarcinoma with clear circumferential margins and due to the nature of stratified columnar epithelium (figure 2) and high CA 19.9, it was considered to be of pancreatic or biliary origin.

This conclusion was not entirely satisfactory due to the lack of specificity of CA 19-9 as a mucinous tumour marker. Also, the CT scans and operative findings were reviewed retrospectively and it was confirmed that the cyst was not involved with the pancreatic or biliary tree. It was thus concluded that this was adenocarcinomatous change within an enterogenous cyst as there was smooth muscle lining the walls with cytokeratins CK20 and CK7 both positive, suggesting a fore-gut origin. Figure 2 is a picture of the histological slide. Also, there was clear carcinomatous change in a plaque within the cyst wall with no penetration from the outer lining described, suggesting no external origin of the malignancy. The patient was to be given Fluoropyrimidine and oxaliplatin following recovery from surgery.

**Figure 3:**
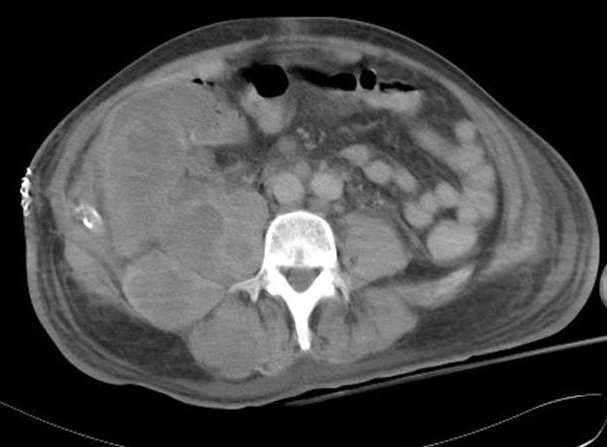
Computed Tomography image showing multiple recurrent lesions in the psoas muscle.

With such a rare condition there is no evidence base for the role of adjuvant chemotherapy. A discussion was had with the patient regarding the possible benefits and risks of adjuvant chemotherapy. In view of the immunohistochemical findings the use of a regimen containing a fluoropyrimidine and oxaliplatin was recommended. Within 3 weeks of surgical excision, the patient developed back pain radiating down the right lower limb. He also had an intermittent fever. He was admitted to hospital and commenced on intravenous antibiotic therapy with a working diagnosis of an infected psoas abscess. However, serial blood cultures grew no organisms. A CT scan demonstrated multiple lesions in the right psoas muscle (Figure 3). Two sequential CT guided fine needle aspirations of these lesions were performed and both revealed cellular appearances suspicious of metastatic adenocarcinoma. The immunohistochemical profile performed on these aspirates was not conclusive. Consequently a laparotomy and open biopsy of these lesions was performed. Unfortunately, multiple peritoneal deposits were apparent at laparotomy. The biopsy demonstrated metastatic adenocarcinoma similar to the original enterogenous cyst with the same differential cytokeratin profile. The Ki67 proliferation fraction was over 80%. His performance status deteriorated greatly, thus preventing the administration of palliative chemotherapy. He died of metastatic disease, 6 weeks following resection of the cyst.

## Discussion

Enterogenous cysts (duplication cysts) are rare. Their presence have been described in the pericardium [6], central nervous system[7], testis [8] and mediastinum [9]. They can also occur anywhere within the gastro-intestinal tract, although they are most commonly found in the small intestine [4]. They tend to present in infancy or early childhood [4,6,9].

Dardik *et al*., [2] described pathological criteria for the diagnosis of gastrointestinal enterogenous cysts. These include the presence of alimentary mucous membrane lining, a smooth muscle coat and intimate attachment to some part of the gastro-intestinal tract. However, cysts with respiratory epithelial lining and no attachment to the gastro-intestinal tract have been described [4,10]. The current article demonstrates a retroperitoneal enterogenous cyst that also does not conform to Dardik's original criteria. Therefore, these criteria should be amended to include that such cysts need not arise from any particular organ.

The embryogenesis of enterogenous cysts have included intra-uterine volvulus resulting in ischaemia and infarction, persistence of an intra-uterine diverticulum and failure of complete vacuolisation of the solid alimentary tract [2-4]. This case represents the third report in the literature of a retroperitoneal enterogenous cyst with adenocarcinomatous changes [4,5], and only the second of such a cyst not arising from any anatomical structure[4].

The two CT scans (the first in 2002, the second in 2006) initially reported an intimate relationship of the cyst with the liver. Initially, radiographic and clinical signs suggested a simple benign liver cyst resulting in the patient's discharge. However, at surgery 4 years later, the cyst did not appear to be arising from any organ and retrospective review of the CT scans concurred with this finding. Previously, enterogenous cysts have been defined as arising from a gastro-intestinal organ[2], although one report has described such a cyst with no relationship to another organ[4,10], which may alter this definition.

Duplication cysts of the gastro-intestinal tract, specifically arising from the rectum, have been described with in-situ adenocarcinoma [11-14]. In these cases many have been successfully excised surgically. However, only one case in the literature has been described arising from no organ. Published cases re-iterate the lack of understanding with regard to adjuvant chemo- or radio-therapy. Also, the cases in the literature do not demonstrate the aggressive malignant potential seen in the current article.

This case illustrates the difficulties in making the diagnosis and the dangers of leaving such cysts untreated.

Histology revealed invasive in-situ adenocarcinoma, which has been previously described [4]. Immunohistochemistry of the epithelium showed that cytokeratins 7 (CK7) and 20 (CK20) were both positive. Classically, adenocarcinoma of the lung, breast ovary and omentum are CK7 positive and CK20 negative, while adenocarcinoma of colorectal origin tends to have the opposite (CK7 negative and CK20 positive) [4,15].

In view of the differential cytokeratin profile in this case (CK 7 and 20 positive) suggesting a fore gut origin, an adjuvant chemotherapy regimen comprising a fluoropyrimidine and oxaliplatin was recommended. Due to the rarity of such cases no direct evidence regarding adjuvant systemic therapy could be applied. Unfortunately, the condition of this patient deteriorated rapidly and he did not receive chemotherapy.

The aggressive nature of this type of cyst has not previously been described. The cyst had remained dormant for at least 4 years. The high levels of CA19.9 related to the cyst may be a marker of its aggressive nature. The authors do not know if tumour markers should be measured routinely in cases similar to this.

Following surgical excision the patient developed rapidly progressive metastatic disease. Although the patient died before receiving chemotherapy, the decision to treat with adjuvant therapy was made since the cyst had ruptured during surgery. Although its role remains to be defined the aggressive malignant potential demonstrated in this article suggests that post-surgical adjuvant therapy should be considered.

## Conclusion

This represents the third case in the literature of adenocarcinoma arising within a retroperitoneal enterogenous cyst. It is also the second case of such a cyst not associated with any anatomical structures and resulted in death due to aggressive uncontrollable malignant disease following excision. Cases like these must be reported in order to build a profile of clinical and pathological presentations of these rare cysts. This case demonstrates the difficulties in make a diagnosis, the importance of a multi-disciplinary approach and raises further questions regarding post-operative treatment with chemotherapy.

## Competing interests

The author(s) declare that they have no competing interests.

## Authors' contributions

JTLprepared the draft manuscript. RLJ, NDK, SdS and JGW helped in preparation of the manuscript

GM and NM edited the final version

All authors read and approved the final manuscript.
